# Cardiovascular Disease, the Nitric Oxide Pathway and Risk of Cognitive Impairment and Dementia

**DOI:** 10.1007/s11886-017-0898-y

**Published:** 2017-08-11

**Authors:** Blossom C. M. Stephan, Stephanie L. Harrison, Hannah A. D. Keage, Abrar Babateen, Louise Robinson, Mario Siervo

**Affiliations:** 1 Institute of Health and Society and Newcastle University Institute for Ageing, Newcastle University, Newcastle Biomedical Research Building, Campus for Ageing and Vitality, Newcastle upon Tyne,, NE4 5PL UK; 2 Department of Rehabilitation, Aged and Extended Care, Faculty of Medicine, Nursing and Health Sciences, School of Health Sciences, Flinders University, Adelaide, Australia; 3 Cognitive Ageing and Impairment Neurosciences Laboratory, School of Psychology, Social Work and Social Policy, University of South Australia, Adelaide, Australia; 4 Institute of Cellular Medicine, Newcastle University, Newcastle Biomedical Research Building, Campus for Ageing and Vitality, Newcastle upon Tyne,, NE4 5PL UK; 5 Faculty of Applied Medical Sciences, Clinical Nutrition Department, Umm Al-Qura University, Makkah, Saudi Arabia

**Keywords:** Cardiovascular disease, Cognitive impairment, Dementia, Endothelial function, Nitric oxide

## Abstract

**Purpose of Review:**

In this review, we summarise the evidence on the association between cardiovascular disease (CVD) and cognitive impairment and explore the role of the nitric oxide (NO) pathway as a causal mechanism.

**Recent Findings:**

Evidence from epidemiological studies suggests that the presence of CVD and its risk factors in midlife is associated with an increased risk of later life cognitive impairment and dementia. It is unclear what is driving this association but risk may be conveyed via an increase in neurodegeneration (e.g. amyloid deposition), vascular changes (e.g. small vessel disease) and mechanistically due to increased levels of oxidative stress and inflammation as well as changes in NO bioavailability.

**Summary:**

CVDs and dementia are major challenges to global health worldwide. The NO pathway may be a promising biological candidate for future studies focused on reducing not only CVD but also risk of cognitive decline and dementia.

## Introduction

Midlife cardiovascular disease (CVD) and vascular risk factors have been consistently associated with an increased risk of later life cognitive impairment and dementia [1–3]. The underlying mechanisms explaining how CVDs and their risk factors negatively affect cognitive function are complex, remain unclear and will likely vary depending on the type, age of onset, duration and severity of disease and co-occurring risk factors such as low educational attainment and disease-related co-morbidity [4]. In addition to neurovascular (e.g. small vessel disease) and neuropathological (e.g. amyloid disposition) processes, other biological pathways are thought to be involved, such as the nitric oxide (NO), inflammatory (e.g. interleukin-6) and oxidative stress pathways. Each of these processes and the NO pathway have been implicated in the pathophysiology of CVD, vascular risk factors, cerebrovascular disease (e.g. stroke), cognitive impairment and dementia. However, the direction of causality is yet to be established.

The aim of this review is to firstly provide an overview of studies investigating the link between CVD and cognitive function including risk of dementia focusing on midlife to later life; and, secondly, discuss how the NO pathway can explain the risk for dementia conferred by CVD and its risk factors. Determining the mediating effect of the NO pathway between CVD and cognitive impairment and dementia is important to (1) identify and validate sensitive biomarkers to discriminate individuals at greatest risk of future cognitive decline and dementia; (2) test the efficacy of targeted nutritional interventions enhancing NO availability and whether these exert downstream positive effects on brain metabolic and vascular regulation; and, (3) inform dietary and lifestyle prevention programmes to concomitantly reduce incidence of CVD and dementia in older aged populations.

## CVD, Cognitive Function and Dementia

Systematic reviews consistently report impairment in cognitive function and an increased risk of dementia (including all-cause, Alzheimer’s disease and vascular dementia) in individuals with a history of CVD including coronary heart disease, heart failure and atrial fibrillation [5–8]. In addition, vascular risk factors such as hypertension, hyperlipidaemia, diabetes, excess adiposity, metabolic syndrome and physical inactivity, as well as CVD risk prediction models (e.g. the Framingham CVD and coronary heart disease risk scores), have been associated with cognitive decline and future risk of cognitive impairment and dementia in older aged populations [9••, 10–12]. Although general cognitive impairments have been shown in relation to CVD and vascular risk factors, deficits appear initially and more predominantly within attention, executive function (i.e. higher level cognitive skills associated with mental control and self-regulation such as planning, decision making and inhibition) and processing speed domains [13–15]. Where associations have not been reported, studies have been found to be under-powered for cognitive/dementia outcomes, have used limited neuropsychological tests restricted to a single or few cognitive domains and generally have had short follow-up times [9••]. The results may also depend on the characteristics of the study population (e.g. age, sex, ethnicity, disease severity), follow-up length, whether disease is controlled (pharmacological vs. non-pharmacological), presence of disease-related co-morbidity and population sampling (i.e. clinical vs. population based).

Indeed, the role of CVD and its risk factors in the development of cognitive impairment and dementia becomes less prominent, and potentially protective, with advancing age. Obesity (measured using the body mass index or waist-to-hip ratio) [16–18], hypertension [2], the metabolic syndrome [19] and hypercholesterolaemia [20–22] have been associated with non-significant or even protective effects on risk of cognitive decline and dementia in the very old population, defined as persons aged ≥85 years. Each of these conditions may have a distinct biological substrate explaining the reduced risk of cognitive impairment and dementia in this age group. For example, the obesity paradox could be explained by the role of adipose tissue in body weight regulation and as an energy provider for costly physiological processes (e.g. immune function, glomerular filtration or haematopoiesis) [23]. These functions may become vital at later stages of life when the capacity of frail bodies to cope with stressors and adversities is diminished and all available resources are required to help maintain physical and mental health [24]. The specific mechanisms that could explain the hypertension findings are still uncertain, but a higher blood pressure may maintain brain tissue vascular perfusion and a potential drop in blood pressure could be expected in individuals developing dementia due to dysregulation of autonomic regulatory mechanisms controlling vascular tone [25, 26]. Regarding the metabolic syndrome, the lack of an association in very old age groups could be due to how the metabolic syndrome is measured (i.e. cut-offs may not be age sensitive) and the fact that it is not a single entity but rather an amalgamation of several cardio-metabolic risk factors [27]. Regarding cholesterol, higher levels may be protective at very old age since cholesterol is an essential component in the structure of the myelin sheath of neurons, key for efficient transmission of nerve impulses and brain functioning [28, 29].

These results highlight that CVD and its risk factors are associated with cognitive impairment and dementia in middle-aged and young-old (i.e. 65–84 years of age) individuals. Therefore, it seems that the timing of disease (e.g. mid versus later life) and sample characteristics (e.g. age) are important in determining the strength as well as the direction (i.e. risk versus protective) of any association. Additional studies are needed to understand whether the tracking of CVD and its risk factors across the life course (i.e. duration of disease) and timing when the condition occurs influence the pattern of associations between CVD and its risk factors with cognitive function and dementia.

## Structural and Functional Brain Changes Associated with CVD and Vascular Risk Factors: Links to Cognitive Impairment and Dementia

CVD can alter the brain structure and functioning including an increase in white matter lesions, small vessel disease, micro-bleeds, cerebral infarcts, grey matter atrophy and regional structural alterations (e.g. in the hippocampus), in addition to cerebral hypoperfusion and increased amyloid disposition [30, 31]. Each is a potential pathological mediator in the CVD-cognition pathway involved in the initiation and progression of cognitive symptoms [3]. Further, a recent review of brain changes associated with vascular risk factors including hypertension, obesity, hyperlipidaemia, diabetes and the metabolic syndrome also found evidence of brain structural and functional changes even in asymptomatic individuals that have been linked to cognitive function [32]. Findings from autopsy studies suggest additive or synergistic links between Alzheimer’s disease and vascular (i.e. cerebral amyloid angiopathy and small vessel disease) pathologies on the influence and promotion of cognitive impairment and risk of dementia in older aged populations [33]. Taken together, it emerges that an integrated, complex network of vascular, metabolic and neural mechanisms regulating cerebral blood flow and cognitive processes are likely to be important components in the pathophysiology underlying cognitive impairment and dementia in the presence of vascular disease.

## Inflammation and Oxidative Stress

In all age groups, including the very old, a significant and increased risk of cognitive decline and dementia has been associated with high inflammation (e.g. C-reactive protein and interleukin-6) and oxidative stress (e.g. homocysteine) levels [10, 34]. Both oxidative stress and inflammation are closely connected to the pathophysiology of CVD and neurological diseases such as dementia [35, 36]. Regarding cardiovascular health, higher levels of oxidative stress and inflammation have been linked to the pathogenesis of atherosclerosis [37]. In relation to the brain, higher levels of oxidative stress and inflammation have been linked to impaired cellular function which may have direct effects on neuronal structure and integrity [38]. Further, inflammation is thought to be important in neurodegeneration, as a consequence as well as contributor to the development of some of the classic hallmarks of Alzheimer’s disease pathology such as amyloid-beta plaques [39]. Oxidative stress and inflammation therefore contribute to the pathogenesis of CVDs and dementia and a key intermediate mechanistic link between these two conditions and its risk factors maybe be represented by the NO pathway given the pleiotropic roles of NO in the regulation of vascular, metabolic, immune and cognitive functions [40].

## Physiological Roles of Nitric Oxide Production in Brain Function

NO is a reactive gas secreted in endothelial cells by the endothelial isoform of the enzyme NO synthase and is tonically released to control systemic vascular tone (i.e. arterial tone, cardiac output and venous capacitance) and platelet aggregation [41]. The vascular endothelium represents a physical and functional interface between circulating blood corpuscolate, fluid blood components (e.g. nutrients) and tissue metabolic processes [42]. Damage to the vascular endothelium is considered as a key pathogenetic element in the onset of atherosclerosis and CVD [43].

**Fig. 1:**
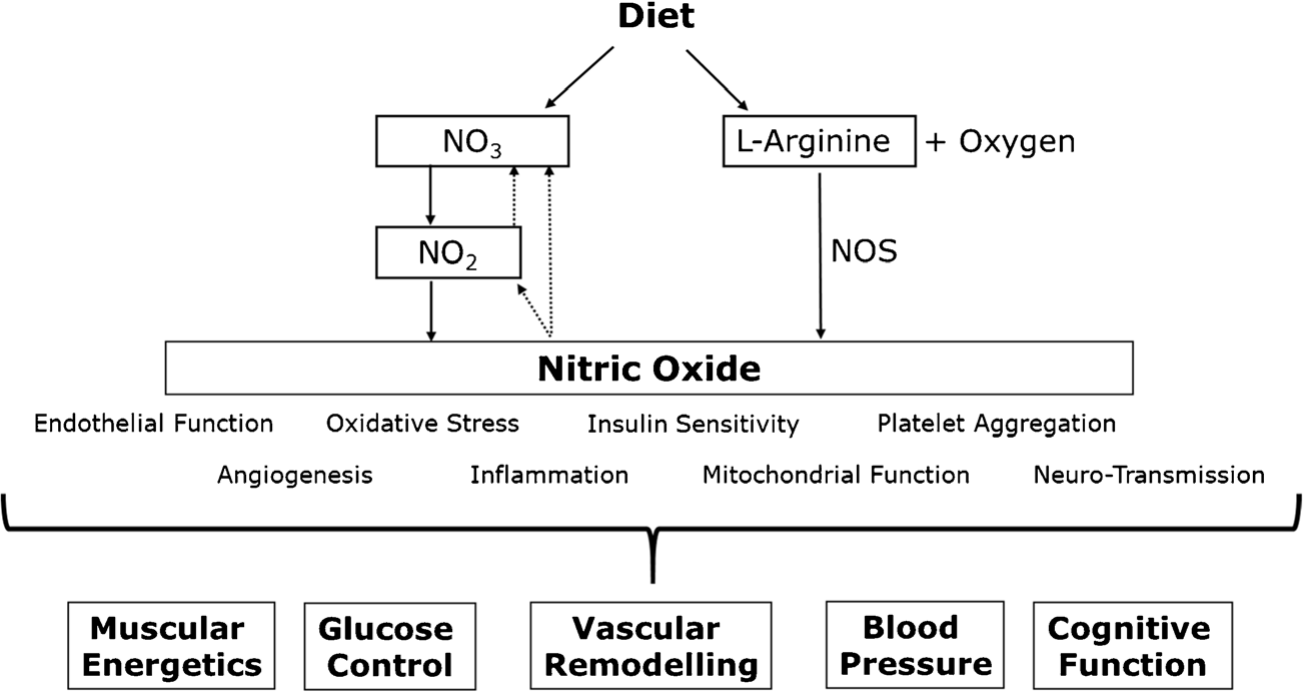
Pathways involved in the synthesis of nitric oxide and downstream physiological effects. *NO*_*3*_ nitrate, *NO*_*2*_ nitrite, *NOS* nitric oxide synthase. (Modified from Jones AM. Sports Medicine (Auckland, New Zealand). 2014; 44 (Suppl 1):35–45) [45]

NO synthase converts l-arginine into an equimolar amount of NO and citrulline (enzymatic synthetic pathway) [41]. NO can also be generated by an alternative non-enzymatic pathway. This involves a two-step reduction of inorganic nitrate into nitrite by oral bacteria (first step) and further reduction of nitrite into NO in the stomach or vascular periphery by the low gastric pH and reductase activity of selected enzymes (second reducing step) [44]. A description of the NO synthetic pathways and pleiotropic effects on physiological functions is provided in Fig. 1.

The production of NO in the brain and its role in the control of neuronal functions has been comprehensively investigated in animal studies [46]. However, little evidence on the role of NO in brain function in humans exists. In a small number of studies, it has been demonstrated that NO is involved in learning and memory processes [47]. For example, NO donors (molsidomine, S-nitroso-*N*-acetylpenicillamine and sodium nitro-prussiate) and l-arginine have been found to help increase NO availability in the brain resulting in improvements in learning and memory [48, 49]. In addition, methyl analogues of l-arginine (i.e. asymmetric dimethylarginine, ADMA or nitro-l-arginine methyl ester), which are known to inhibit NO synthesis, have been associated with cognitive impairment in older adults with and without dementia [50, 51].

The beneficial effects of NO on brain function (e.g. learning and memory) appear to be mediated by several mechanisms. These include nitrosylation of *N*-methyl-*D*-aspartic acid (NMDA) and augmentation of excitability via modulation of voltage-gated potassium channels, which can mediate a calcium-dependent activation of neuronal NO synthase and downstream canonical cyclic guanosine monophosphate (cGMP)-mediated signalling. NO also regulates other pathways via the post-translational modification (S-nitrosylation and 3-nitrotyrosination) of proteins involved in synaptic transmission and intracellular trafficking [52]. However, evidence also suggests that high NO generation could lead to abnormal protein modifications and impact on the pathogenesis of various neurodegenerative diseases, including Parkinson’s disease and Alzheimer’s disease [53••]. Hence, a better understanding of the physiological role of NO on brain function and identification of the specific regulatory mechanisms may represent an important diagnostic and therapeutic strategy for the maintenance of cognitive function.

## Endothelial Dysfunction, NO and Cognitive Impairment

Endothelial function is closely linked to the control of cerebrovascular reactivity, which is essential for creating a favourable environment for neurons, by maintaining energy-dependent processes and removing metabolic waste [54–56]. It has been suggested that impaired cerebrovascular reactivity is not only related to CVDs, but can also be associated with dementia [57]. Studies have explored the association between physiological (i.e. flow-mediated dilation and pulse wave velocity) and circulating biomarkers of endothelial function and NO bioavailability (i.e. nitrate, nitrite, ADMA) with cognitive impairment [51, 58–61]. Flow-mediated dilation was found to be lower in patients with Alzheimer’s disease compared to that of controls and was significantly correlated directly with global cognitive function (Mini Mental State Examination scores) and Clinical Dementia Rating scores (inverse association) [62]. Similarly, pulse wave velocity has been found to be significantly higher in Alzheimer’s disease patients compared to controls without cognitive impairment [63]. Patients with Alzheimer’s disease have also been found to have higher plasma concentrations of ADMA and lower levels of nitrate compared to healthy controls [51, 64]. In contrast, two studies have reported lower ADMA concentrations in cerebrospinal fluid of Alzheimer’s disease patients compared to healthy controls [65, 66] whereas one study found no significant differences between Alzheimer’s disease patients and age-matched control subjects [67]. Inconsistencies in findings could be related to differences in the stages of dementia across studies [67] and the degree of methylation of arginine residues in the brain due to a reduction of S-adenosyl-l-methionine [68]. S-Adenosyl-l-methionine concentrations are decreased in cerebrospinal fluid of Alzheimer’s disease patients which could have an effect on the generation and release of ADMA in the brain [69]. The net effect of this decrease in ADMA concentrations is an increased generation of NO in the brain which could be more susceptible to react with oxidative species and generate peroxynitrite, thus contributing to the pathogenesis of Alzheimer’s disease [70, 71]. Together, these results highlight the role of endothelial dysfunction in the pathophysiology of dementia and suggest that physiological markers of endothelial dysfunction are more closely associated with cognition compared to circulating biomarkers of NO.

It has also been suggested that impaired NO and endothelial dysfunction could be associated with cognitive impairment, and the development of Alzheimer’s disease, possibly due to dysfunction of cerebral blood flow and reduced oxygen supply to the brain [72]. Disruption of the neurovascular function could therefore be considered as a major risk factor for cerebral vascular deregulation, which could be affected by compromised NO activity. Manukhina et al. found that a reduced NO production in rats, following the administration of the NO synthase inhibitor NG-nitro-l-arginine methyl ester (L-NAME), increased the harmful effects of beta-amyloid and was linked to memory deficits like those observed in Alzheimer’s disease, whereas the administration of NO donors showed a protective effect on beta-amyloid deposition [73]. As beta-amyloid is the primary component of the extracellular plaques and is increased in neurodegenerative disease, investigating the association between NO and beta-amyloid might give a better understanding of how vascular function is linked to neuronal damage.

## NO-Targeted Nutritional Interventions

The biosynthesis of NO is highly dependent on arginine and inorganic nitrate intake since they are the main substrates for the enzymatic and non-enzymatic pathways, respectively. In addition, NO synthesis can be enhanced by the presence or activity of other nutrients such as vitamin C, polyphenols or polyunsaturated fatty acids [41, 44]. Arginine supplementation has been shown to improve cognitive function and cerebral blood flow in 16 older aged adults (mean age 79 years) [74]. However, more robust and larger clinical trials are needed to confirm these preliminary results. Dietary nitrate supplementation has also been associated with significant benefits on blood pressure and endothelial function [75•, 76]. A few studies have investigated the effect of dietary nitrate on cerebral blood flow, and a summary of these studies is described in Table 1. Overall, dietary nitrate has been found to enhance cerebral blood flow in humans of different ages and has been associated with improved cognitive processes [77–79]. However, studies are characterised by a short duration, small sample size and have restricted recruitment to healthy individuals with normal cognitive function. Therefore, it remains to be established whether nitrate supplementation over longer time periods influences cognitive function in older individuals (≥60 years) with cognitive impairment.

**Table 1:** Examples of human studies investigating the effect of inorganic nitrate on cerebral blood flow (CBF)

Reference	Participants	Study design	Measures	Dietary intervention/period	Main findings
Presley et al. 2011 [77]	16 healthy older individuals	RCT, crossover	CBF	High nitrate diet vs. low nitrate diet for 2 days	CBF increased with high-nitrate diet
Bond et al. 2013 [78]	12 healthy adults	RCT, crossover	CVRI, BP, TVR and BP measured at rest and at two exercise workloads	500-ml BJ or placebo (orange juice)/single dose	CVRI was decreased after single beetroot juice treatment at rest and at submaximal exercise workloads
Wightman et al. 2015 [79]	40 healthy adults	RCT, double-blind, placebo, parallel	NIRS, cognitive tasks (including 3s and 7s verbal series), BP, HR	450-ml BJ or placebo drink with negligible nitrate content/150 min	Nitrate improved CBF and performance on 3s serial subtractions test but not others

*BJ* beetroot juice, *BP* blood pressure, *CBF* cerebral blood flow, *CVRI* cerebrovascular resistance index, *HR* heart rate, *NIRS* near-infrared spectroscopy, *RCT* randomised clinical trial, *TVR* total vascular resistance

## Implications for Research

The findings highlight that CVDs and vascular risk factors are major risks for the onset of cognitive decline and dementia. However, timing of the disease matters as the association varies with age. Further, while there is some preliminary evidence on the association between biomarkers of NO availability and cognitive processes in humans, most of research is still limited to animal models. However, these studies have demonstrated a role of NO-related mechanisms in the regulation of cognitive processes alongside the established effects on blood flow and cellular metabolism. These data can therefore prompt the development of research hypotheses in this area, such as to explore the association in longitudinal studies between physiological (i.e. flow-mediated dilation and pulse wave velocity) and biochemical (nitrate, nitrite, ADMA) biomarkers with prospective changes in cognitive function and risk of incident dementia. Positive results may have important diagnostic implications for the development of risk prediction and screening models for the early identification of individuals at risk of and with cognitive decline and dementia. Further, two nutrients (i.e. arginine and inorganic nitrate) are the precursors of NO in the enzymatic and non-enzymatic synthetic pathways and therefore dietary patterns (e.g. Mediterranean diet, DASH diet) or foods rich in these nutrients (e.g. lettuce, rocket, cabbage, eggs, beetroot, nuts) may be examples of targeted nutritional interventions aimed at increasing NO bioavailability and potentially reducing risk of CVD as well as improving cognitive function. However, the effects derived from the supplementation of these nutrients on cognitive function have not been explored in human clinical trials.

## Conclusions

With a rapidly ageing population, CVDs and dementia are major challenges to global health and future health care provision. A critical characteristic of these trends is that the largest proportion of cases will be in low- and middle-income countries where public health resources and clinical services are limited. Therefore, sensitive and affordable biomarkers for the early identification of CVD and dementia risk are an urgent priority alongside testing effective nutritional and lifestyle preventative and treatment strategies to maintain cognitive function. The NO pathway may be a promising biological candidate to respond directly to these requisites (biomarkers and interventions) and future studies testing the role of NO as a mediator of the association between cardiovascular diseases and cognitive function are awaited.
